# Incidence of suicide within two years of a first diagnosis of depression, anxiety, or mixed anxiety and depression: an exploratory cohort study in primary care using the Clinical Practice Research Datalink

**DOI:** 10.1016/j.eclinm.2025.103441

**Published:** 2025-08-25

**Authors:** James Bailey, Patricia Schartau, Seena Fazel, Irwin Nazareth, Irene Petersen

**Affiliations:** a Department of Primary Care and Population Health, University College London, London, UK; b Department of Psychiatry, University of Oxford, Oxford, UK; c Department of Clinical Epidemiology, Aarhus University, Denmark

**Keywords:** Suicide, Primary care, Incidence, Depression, Anxiety, Prognosis

## Abstract

**Background:**

The risk of suicide following first diagnosis of depression or anxiety in primary care is uncertain. We investigated suicide incidence within two years of a first diagnosis of depression, anxiety, or mixed anxiety and depression in primary care, analysing variation by diagnosis, age, sex, and social deprivation at lower layer Super Output Area level.

**Methods:**

We conducted a cohort study using the Clinical Practice Research Datalink, a large primary care electronic health records database, linked to mortality records. Adults with their first diagnosis of depression, anxiety, or mixed anxiety and depression between 1 January 1999 and 31 December 2018 were included. The outcome was suicides per 100,000 person-years at risk (PYAR) within two years of diagnosis. Adjusted incidence rate ratios (aIRR) were calculated using Poisson regression.

**Findings:**

Among 1,454,102 individuals diagnosed there were 1439 suicides within two years of diagnosis. Rates were higher in men across all cohorts. The highest rate for men was after depression diagnosis: 115·85 per 100,000 PYAR (95% confidence interval [CI] 107·60–124·58), five times higher than women: aIRR 4·99 (95% CI 4·29–5·79). For women the highest rate was after a mixed diagnosis: 27·73 per 100,000 PYAR (95% CI 21·70–34·92). The highest rate across all groups was seen in men aged 70+ after a mixed diagnosis: 156·43 per 100,000 PYAR (95% CI 89·41–254·03). There was no association between rate and deprivation.

**Interpretation:**

Suicide rates within two years of a diagnosis were higher than the UK general population rate as reported by the Office for National Statistics. Men consistently exhibited higher rates, with men aged 70 and over diagnosed with mixed anxiety and depression experiencing the highest rate. Women aged 50–59 with a first diagnosis of anxiety had over three times the rate of those aged 18–29 at diagnosis. These findings align with findings from other settings and add to the literature by quantifying the effects in primary care. Further analysis is required, particularly for older men and middle-aged women with anxiety-related conditions.

**Funding:**

James Bailey is funded by a 10.13039/501100000272National Institute for Health and Care Research Doctoral Fellowship [NIHR302551].

**Research in context**

**Evidence before this study**

We searched EMBASE on 1 July 2024, for publications since database inception with no language restriction using the terms (“suici∗”) and (“depress∗” or “anxi∗” or “common mental disorder∗”) and (“rate” or “incidence” or “prevalence”) and (“diagnosis”) and identified 5538 articles. Several studies estimated the rate of suicide after a diagnosis of a common mental disorder such as depression or anxiety in people attending psychiatric outpatient clinics or on after discharge from an inpatient mental health unit as well as several psychological autopsy studies.

**Added value of this study**

This study adds to the body of literature estimating rates of suicide associated with mental health diagnoses, and provides a primary care perspective. We have identified that men have up to five times the rate of suicide after a first diagnosis compared to women and that the age at first diagnosis has a significant impact on rates. We have described that the high rate of suicide within two years of a diagnosis of an anxiety-related condition in men aged 70 and over may be a driving factor behind the high suicide rate seen in this group at the population level. This exploratory work highlights two groups in particular, men aged 70 and over and women aged 50–59 who, if our findings are corroborated, warrant further investigation to understand their experiences before and after diagnosis.

**Implications of all the available evidence**

Estimates of suicide rates for people diagnosed with common mental disorders varies by setting. The higher rate of suicide after discharge from mental health units is well documented and has received particular attention from both clinicians and policymakers. However, the majority of people who die from suicide in the United Kingdom are not known to secondary mental health services. Understanding the experiences of people presenting for the first time to primary care informs clinicians and policymakers of how to improve assessment of risk and engage at an early stage, thereby giving people the support they need at a critical point in their lives.

## Introduction

Suicide is a major global health concern, causing over 700,000 deaths annually.^1^ In England and Wales, there were 6069 suicides in 2023—the highest number since 2000—with significant differences between men (17·4 per 100,000) and women (5·7 per 100,000).^2^ Current estimates of suicide rates among individuals with mental health diagnoses primarily come from data collected from outpatient and inpatient mental health settings linked to hospitals (secondary mental health services).^3^^,^^4^ However, under the UK's National Health Services (NHS), access to secondary mental health services requires a referral, usually from a general practitioner (GP).^5^ There are only certain circumstances where the National Institute for Health and Care Excellence (NICE), the main public body that produces evidence-based clinical guidelines for the UK, suggests referral on to secondary mental health services after a diagnosis of depression or anxiety. For depression, referral to secondary mental health services is recommended in the event of psychotic symptoms, chronic depressive symptoms which significantly impair personal and/or social function that have not responded to treatment, and those with a diagnosis of depression combined with a personality disorder.^5^ There are similar referral criteria in the guidelines for anxiety.^6^ This contrasts with diagnoses such as Post-Traumatic Stress Disorder or presentations which include symptoms of psychosis where early referral to secondary mental health services is advised.^7^^,^^8^

The National Confidential Inquiry into Suicide and Safety reports that only a quarter of people who die by suicide have been seen by secondary mental health services in the year before death.^9^ In contrast, about 90% have consulted their GP in the year before their death, often regarding mental health issues and average ten consultations in that year.^10^^,^^11^ Understanding suicide risk among primary care attendees is essential for identifying those most at risk and informing initial management strategies.

This study aims to describe suicide rates within two years of a first primary care diagnosis of depression, anxiety, or mixed anxiety and depression, and to analyse variations by age at diagnosis, sex, and deprivation.

## Methods

We conducted an exploratory cohort study using data from general practices in England contributing to the Clinical Practice Research Datalink (CPRD) Aurum dataset, which contains routinely collected electronic health records representative of the wider population and included the data of over 19 million individuals by the end point of the study.^12^ CPRD Aurum includes diagnoses, consultations, test results, referrals, demographic data, problem lists, and prescriptions and is linked to the Office for National Statistics (ONS) Death Registration data for cause of death information. The Index of Multiple Deprivation (IMD) is linked at a patient level to CPRD and is an aggregate measure of deprivation split into quintiles. This aggregate measure is the weighted sum of deprivation domains consisting of income, employment, education and skills, health, housing, crime, access to services, and living environment.^13^

Individuals were eligible for inclusion if they were aged 18 or over at diagnosis, permanently registered at their GP practice, had at least six months of registration time prior to diagnosis (to avoid misclassifying past psychiatric history as new diagnoses), and had no previous diagnostic codes for depression, anxiety, or mixed anxiety and depression. We did not exclude individuals based on any previous diagnosis or event other than depression, anxiety, or mixed anxiety and depression. Eligible individuals entered their respective cohort if their first diagnosis of depression, anxiety, or mixed anxiety and depression (index diagnosis) occurred between 1 January 1999 and 31 December 2018. No records provided by CPRD had missing sex or date of birth. A total of 2724 people were excluded from the analysis: the 35 people with sex coded as “indeterminate” (an insufficient number to report rates for whilst maintaining anonymity) and the 2689 people with missing IMD. Diagnostic codes were reviewed by JB and IP and chosen where they suggested a new diagnosis of depression, anxiety, or mixed anxiety and depression (Appendix 1). Mixed anxiety and depression was defined as either a diagnostic code specific to this condition or if more than one diagnosis was coded on the index diagnosis date. This reflects how mixed anxiety and depression as a diagnosis is applied in UK primary care. Individuals were followed up until censored at the date of death, deregistration, or two years after their index diagnosis. Participants were only included in one diagnostic cohort, based on their earliest diagnosis, and could not subsequently enter a second cohort.

The outcome was death from suicide within two years of the index diagnosis, defined using ICD-10 or ICD-9 codes for self-inflicted death in linked ONS Death Registration data, aligned with the ONS methodology for reporting national suicide statistics (Appendix 2).^14^, ^15^, ^16^ A sensitivity analysis including codes for accidental death was performed (Appendix 3).

Suicide rates per 100,000 person-years at risk (PYAR) were calculated with 95% confidence intervals (CI). Incidence rate ratios (aIRRs) were calculated using Poisson regression and adjusted for age group and IMD. Results were stratified by sex and diagnosis. Sex, age, and Index of Multiple Deprivation (IMD) were considered as categorical variables. Age at diagnosis was categorised into: 18–29, 30–39, 40–49, 50–59, 60–69, and 70+ years to capture potential non-linear relationships with suicide rate. The analysis was conducted in line with the protocol submitted to CPRD, this is publicly available through their bibliography. Statistical analyses were performed using Python (version 3·10·13) with the statsmodels (version 0·14·2).

### PPIE statement

A patient and public involvement and engagement group met and reviewed the plan for this work as part of the lead author's Doctoral Fellowship.

### Ethics approval

Ethical approval for work using CPRD has already been granted (East Midlands - Derby Research Ethics Committee, 21/EM/0265, 10 January 2022). Scientific approval through CPRD's Research Data Governance process, overseen by their Expert Review Committees and Central Advisory committee was sought via the CPRD electronic Research Applications Portal. Scientific approval was granted under protocol number 23_002609.

### Role of the funding source

This work was funded by James Bailey's National Institute for Health and Care Research's Doctoral Fellowship Award [NIHR302551].

## Results

Between 1 January 1999 and 31 December 2018, 1,454,102 individuals had a first diagnosis of depression (n = 883,598), anxiety (n = 337,755) or mixed anxiety and depression (n = 232,749) (Table 1), collectively contributing a total of 2,670,750 person-years. There was a total of 888,673 women (61·11%) with a median age of 39 (IQR 28–54) and 565,429 men (38·89%) with a median age of 41 (IQR 29–55). Broadly, individuals were evenly distributed across quintiles of deprivation. Within two years of diagnosis, 1439 individuals (0·1%) died by suicide, comprising 1083 men (75·3%) and 356 women (24·7%). Of those who died from suicide 3·5% of men and 4·5% of women had a code for “suicide attempt” or self-harm prior to their first diagnosis of depression, anxiety, or mixed anxiety and depression. Men had higher suicide rates than women across all diagnostic cohorts. The highest rate for men was after a diagnosis of depression: 115·85 per 100,000 PYAR (95% CI 107·60–124·58). For women, the highest rate was after a diagnosis of mixed anxiety and depression: 27·73 per 100,000 PYAR (95% CI 21·70–34·92). The highest suicide rates across age groups were observed in men aged 70 and over with mixed anxiety and depression: 156·43 per 100,000 PYAR (95% CI 89·41–254·03) and in women aged 60–69 with mixed anxiety and depression: 51·49 per 100,000 PYAR (95% CI 24·69–94·69).

**Table 1:** Counts of people diagnosed with depression, anxiety, or mixed anxiety and depression between 1999 and 31 December 2018.

	Depression				Anxiety				Mixed anxiety and depression			
	Men		Women		Men		Women		Men		Women	
	n	%	n	%	n	%	n	%	n	%	n	%
**Age**												
Overall	343,787	38·91%	539,811	61·09%	129,711	38·40%	208,044	61·60%	91,931	39·50%	140,818	60·50%
18–29	79,718	23·19%	151,785	28·12%	39,532	30·48%	64,707	31·10%	24,951	27·14%	40,950	29·08%
30–39	70,721	20·57%	122,095	22·62%	27,932	21·53%	40,583	19·51%	21,255	23·12%	30,672	21·78%
40–49	69,080	20·09%	95,436	17·68%	23,151	17·85%	32,439	15·59%	19,183	20·87%	26,254	18·64%
50–59	53,403	15·53%	68,599	12·71%	16,762	12·92%	24,789	11·92%	13,806	15·02%	19,418	13·79%
60–69	31,373	9·13%	37,806	7·00%	10,828	8·35%	18,104	8·70%	6716	7·31%	10,198	7·24%
70+	39,492	11·49%	64,090	11·87%	11,506	8·87%	27,422	13·18%	6020	6·55%	13,326	9·46%
**Index of Multiple Deprivation quintile**												
1 (least deprived)	59,484	17·30%	98,429	18·23%	25,275	19·49%	43,199	20·76%	15,304	16·65%	24,043	17·07%
2	64,589	18·79%	103,621	19·20%	25,804	19·89%	42,815	20·58%	16,788	18·26%	26,621	18·90%
3	64,464	18·75%	102,510	18·99%	24,606	18·97%	39,622	19·05%	17,066	18·56%	26,752	19·00%
4	73,234	21·30%	113,353	21·00%	26,102	20·12%	41,136	19·77%	19,291	20·98%	29,946	21·27%
5 (most deprived)	82,016	23·86%	121,898	22·58%	27,924	21·53%	41,272	19·84%	23,482	25·54%	33,456	23·76%

### Depression

Of the individuals diagnosed with depression, 61·09% were women (Table 1). The median age at diagnosis was 39 (IQR 28–54) for women and 42 (IQR 30–56) for men. A total of 955 people (729 men and 226 women) died by suicide within two years. Men had a suicide rate of 115·85 per 100,000 PYAR (95% CI 107·60–124·58) (Table 2), five times higher than for women (adjusted IRR (aIRR) of 4·99 (95% CI 4·29–5·79)) (Table 3). The median time from diagnosis to suicide within two years was 168·5 days (IQR 47–399) for men and 252·5 days (IQR 88–432·5) for women.

**Table 2:** Suicide rates within two years of a first diagnosis of depression, anxiety, or mixed anxiety and depression per 100,000 person-years at risk.

	Depression				Anxiety				Mixed anxiety and depression			
	Men		Women		Men		Women		Men		Women	
	Rate	(95% CI)	Rate	(95% CI)	Rate	(95% CI)	Rate	(95% CI)	Rate	(95% CI)	Rate	(95% CI)
**Age**												
Overall	115·85	(107·60–124·58)	22·83	(19·95–26·00)	69·34	(59·20–80·73)	15·18	(11·53–19·63)	110·35	(95·14–127·30)	27·73	(21·70–34·92)
18–29	100·60	(84·95–118·31)	21·22	(16·07–27·49)	44·48	(30·42–62·79)	9·65	(4·82–17·28)	68·01	(46·21–96·53)	24·88	(14·75–39·32)
30–39	121·19	(103·03–141·63)	19·45	(14·13–26·11)	48·35	(31·29–71·37)	7·97	(2·93–17·36)	124·43	(92·06–164·51)	15·79	(7·22–29·98)
40–49	128·56	(109·80–149·60)	28·54	(21·31–37·43)	102·62	(74·85–137·31)	14·45	(6·61–27·44)	110·13	(78·68–149·96)	35·87	(21·26–56·70)
50–59	130·56	(109·24–154·83)	27·45	(19·23–38·00)	109·64	(76·37–152·49)	31·49	(17·62–51·94)	129·31	(89·55–180·70)	29·60	(14·78–52·97)
60–69	101·54	(77·30–130·98)	27·94	(17·07–43·15)	59·30	(30·64–103·59)	23·19	(10·01–45·69)	143·27	(84·91–226·43)	51·49	(24·69–94·69)
70+	103·66	(80·33–131·64)	15·42	(8·98–24·68)	86·19	(50·21–137·99)	18·58	(8·49–35·26)	156·43	(89·41–254·03)	25·46	(9·34–55·41)
**Index of Multiple Deprivation quintile**												
1 (least deprived)	128·73	(108·36–151·82)	31·91	(24·23–41·25)	76·57	(53·63–106·01)	16·25	(8·65–27·79)	132·85	(94·01–182·35)	17·89	(7·72–35·25)
2	105·93	(88·17–126·21)	23·08	(16·77–30·99)	73·40	(51·13–102·08)	10·16	(4·39–20·03)	105·89	(72·89–148·72)	24·37	(12·59–42·57)
3	119·74	(100·79–141·21)	16·53	(11·23 - 23·46)	66·18	(44·65–94·48)	23·45	(13·66–37·55)	104·71	(72·08–147·05)	42·82	(26·50–65·45)
4	117·37	(99·73–137·24)	21·28	(15·46–28·57)	70·96	(49·14–99·15)	17·35	(9·24–29·67)	118·05	(85·08–159·57)	25·48	(13·93–42·76)
5 (most deprived)	109·86	(93·74–127·96)	21·94	(16·23–29·01)	60·26	(40·94–85·53)	9·22	(3·71–19·00)	96·53	(69·57–130·49)	27·54	(16·04–44·10)

Rate: Rate of suicide per 100,000 person-years at risk. CI: Confidence interval.

**Table 3:** Adjusted incidence rate ratios for suicide within two years of a first diagnosis of depression, anxiety, or mixed anxiety and depression.

	Depression				Anxiety				Mixed anxiety and depression			
	Men		Women		Men		Women		Men		Women	
	aIRR	(95% CI)	aIRR	(95% CI)	aIRR	(95% CI)	aIRR	(95% CI)	aIRR	(95% CI)	aIRR	(95% CI)
**Age**												
Overall	4·99	(4·29–5·79)	ref.		4·58	(3·39–6·18)	ref.		3·96	(3·02–5·20)	ref.	
18–29	ref.		ref.		ref.		ref.		ref.		ref.	
30–39	1·20	(0·96–1·50)	0·90	(0·60–1·33)	1·08	(0·64–1·82)	0·82	(0·30–2·23)	1·82	(1·16–2·85)	0·64	(0·29–1·42)
40–49	1·28	(1·02–1·59)	1·31	(0·90–1·91)	2·29	(1·46–3·61)	1·50	(0·62–3·62)	1·60	(1·00–2·57)	1·47	(0·76–2·83)
50–59	1·30	(1·03–1·64)	1·26	(0·83–1·92)	2·45	(1·52–3·96)	3·26	(1·49–7·10)	1·88	(1·16–3·07)	1·22	(0·57–2·58)
60–69	1·01	(0·75–1·37)	1·29	(0·77–2·15)	1·32	(0·68–2·57)	2·39	(0·96–5·96)	2·09	(1·17–3·74)	2·12	(0·97–4·59)
70+	1·03	(0·77–1·38)	0·71	(0·41–1·22)	1·92	(1·06–3·46)	1·92	(0·79–4·64)	2·28	(1·24–4·17)	1·05	(0·42–2·65)
**Index of Multiple Deprivation quintile**												
1 (least deprived)	1·17	(0·93–1·46)	1·44	(0·99–2·12)	1·18	(0·73–1·91)	1·60	(0·64–4·02)	1·29	(0·83–2·01)	0·62	(0·27–1·44)
2	0·96	(0·76–1·22)	1·05	(0·70–1·58)	1·16	(0·71–1·88)	1·03	(0·37–2·84)	1·04	(0·66–1·64)	0·85	(0·41–1·79)
3	1·09	(0·87–1·36)	0·75	(0·48–1·18)	1·06	(0·64–1·75)	2·44	(1·01–5·88)	1·04	(0·66–1·64)	1·52	(0·80–2·88)
4	1·07	(0·86–1·33)	0·97	(0·65–1·46)	1·17	(0·72–1·90)	1·86	(0·74–4·66)	1·19	(0·78–1·83)	0·91	(0·45–1·85)
5 (most deprived)	ref.		ref.		ref.		ref.		ref.		ref.	

aIRR: Adjusted incidence rate ratio. CI: Confidence interval.

For men, the highest suicide rate was in those aged 50–59: 130·56 per 100,000 PYAR (95% CI 109·24–154·83) (Table 2), (aIRR of 1·30 (95% CI 1·03–1·64) relative to those aged 18–29) (Table 3). For women, those aged 40–49 had the highest rate 28·54 per 100,000 PYAR (95% CI 21·31–37·43) (Table 2) and those aged 70 and over had the lowest rate 15·42 per 100,000 PYAR (95% CI 8·98–24·68). There was no clear pattern of suicide rates across deprivation quintiles for either sex.

### Anxiety

In the anxiety cohort, 61·60% were women (Table 1). The median age at diagnosis was 39 (IQR 27–57) for women and 39 (IQR 27–53) for men. Within two years, 224 individuals (166 men and 58 women) died by suicide. Men had a suicide rate of 69·34 per 100,000 PYAR (95% CI 59·20–80·73), 4·5 times higher than women: 15·18 per 100,000 PYAR (95% CI 11·53–19·63) (Table 2), (aIRR 4·58 (95% CI 3·39–6·18)) (Table 3). The median time from diagnosis to death from suicide was 168·5 days (IQR 45–391) for men and 197 days (IQR 55–473) for women.

Suicide rates peaked in the 50–59 age group for both men and women (aIRR 2·45 (95% CI 1·52–3·96) for men and 3·26 (95% CI 1·49–7·10) for women, relative to those aged 18–29). No consistent pattern was observed across deprivation quintiles.

### Mixed anxiety and depression

Among those diagnosed with mixed anxiety and depression, 60·50% were women (Table 1). The median age at diagnosis was 39 (IQR 28–53) for women and 39 (IQR 29–52) for men. A total of 260 individuals (188 men and 72 women) died by suicide within two years. Men had a suicide rate of 110·35 per 100,000 PYAR (95% CI 95·14–127·30), nearly four times that of women: 27·73 per 100,000 PYAR (95% CI 21·70–34·92) (Table 2) (aIRR of 3·96 (95% CI 3·02–5·20)) (Table 3). The median time from diagnosis to death from suicide was 197 days (IQR 63·5–422) for men and 223·5 days (IQR 69–454) for women.

For men, suicide rates increased with age, peaking in those aged 70 and over 156·43 per 100,000 PYAR (95% CI 89·41–254·03), aIRR of 2·28 (95% CI 1·24–4·17) relative to those aged 18–29. For women, the highest rate was in the 60–69 age group 51·49 per 100,000 PYAR (95% CI 24·69–94·69), but confidence intervals were wide. As with other cohorts, no clear pattern was observed in suicide rates across deprivation quintiles.

## Discussion

We have presented the incidence of suicide within two years of a first diagnosis of depression, anxiety, or mixed anxiety and depression; describing how the suicide rate varies by sex, age at diagnosis and deprivation. Men had a higher suicide rate than women across all diagnoses. Age affected rates most noticeably in those diagnosed with either anxiety or mixed anxiety and depression. People diagnosed with anxiety alone for the first time in their 50s died from suicide at two to over three times the rate of those diagnosed aged 18–29. Men diagnosed for the first time aged 70 and over with either anxiety alone or mixed anxiety and depression had a suicide rate two times that of those diagnosed for the first time aged 18–29, a pattern not seen for men diagnosed with depression without anxiety. For men with anxiety this formed a “second-peak” pattern and for those diagnosed with mixed anxiety and depression rates increased with age. Men aged 70 and over newly diagnosed with mixed anxiety and depression had the highest rate of suicide of any group (156·43 deaths per 100,000 PYAR). No pattern was seen across IMD quintiles, likely explained by the association between deprivation and both the diagnoses of interest and an increased risk of suicide.

The consistency of coding of common mental disorders, including depression and anxiety is a common limitation in primary care electronic health records. The clinical coding for common mental disorders, such as depression or anxiety, in primary care does not always follow pre-specified criteria and is sometimes avoided even when a diagnosis is considered likely, with some GPs report that they prefer to use symptom rather than diagnostic codes to prevent the assignment of what they consider stigmatising diagnoses, instead assigning diagnostic codes depending on the severity and chronicity of presentation.^17^ Analysing the recording of prescription codes for antidepressant or anxiolytic medications may help identify people who have not had a formal diagnosis but who are being treated for uncoded depression or anxiety. However, we chose not to include this in this study, to better characterise the differences between those with different initial diagnoses as there is significant overlap in pharmacological treatments for depression and anxiety. This study examined suicide rates within two years of an initial diagnosis of depression, anxiety, or mixed anxiety and depression. Consequently, the findings may not be generalisable to long-term suicide risks, nor do they reflect the rates following subsequent clinical encounters, such as a recurrence of depression. This work did not include estimates of rates after other psychiatric diagnoses or diagnostic codes for substance misuse, which are often initially seen by primary care in the UK and carry an increased risk of suicide. This study also did not consider the impact of co-morbid physical health conditions. The interaction between physical health, mental health conditions, and suicide risk requires additional study beyond the scope of this work.

Psychological autopsy studies, where the health records and close contacts of people who have died from suicide are consulted to assign a diagnosis retrospectively, could be considered more consistent in the application of diagnostic criteria for the identification of depression and anxiety. However, rates calculated from psychological autopsy studies are harder for a clinician to contextualise correctly as these studies include people who were not diagnosed before death and may therefore provide different estimates to studying those who have presented to a healthcare provider for diagnosis and management.

Some cases of depression or anxiety will be missed in this study where a diagnosis is made and recorded as either free text or managed under a co-presenting condition. An example of this would be someone who has one appointment where both menopause and depression were discussed but where only menopause was coded.

The rates of suicide per 100,000 PYAR within two years of a first diagnosis of depression, anxiety, or mixed anxiety and depression were higher than the Office for National Statistics' estimated rates for the general population of England in 2008 (the midpoint year of this study): 14·7 deaths per 100,000 men and 4·4 deaths per 100,000 women in that year.^18^ The “second-peak” in suicide rate for men aged 70 and over seen for those with anxiety is reflected in the ONS' mortality reporting, with a second rise in rates for men aged 70 and over, a pattern not seen for women.^19^ This pattern is not seen after a diagnosis of depression and suggests this second peak in men aged 70 and over could be driven in part by anxiety-related conditions. Though following national trends across age groups, the second-peak pattern reported in this study should be interpreted with caution given the breadth of the confidence intervals.

One UK study has described a higher prevalence of anxiety disorders among people who have died from suicide aged over 60 and over compared to those aged under 60, with diagnoses most prevalent in males and in those who lived alone.^20^ This previous study discusses survivor bias as a possible explanation of this difference, suggesting those who are impulsive would be more likely to die at a relatively younger age compared to those with chronic anxiety. A full explanation for this second peak for older men with anxiety but not depression alone requires further investigation. It may be a risk inherent to anxiety presentations in men, how anxiety is managed compared to depression, a reluctance of GPs to code less severe anxiety presentations for men aged 70 and over relative to other groups or common factors that lead to both anxiety and higher suicide rates in men but not in women. The differences in rates seen may also be driven in part by the misclassification of suicides as accidental deaths, which can more commonly occur where the method used leaves a greater ambiguity of intent, such as with overdoses.^21^ The sensitivity analysis in Appendix 3 shows an increase in rates for adults aged 70 and over and particularly for women in this age group when codes for accidental deaths are included. A sensitivity analysis of rates by year of diagnosis revealed no discernible pattern.

The pronounced difference in suicide rate between women diagnosed with anxiety aged 50–59 and those aged 18–29 warrants further attention. The cause of this difference could be related to the same factors that result in the higher rate of suicide seen in men in their forties and fifties, but given the age-related differences for women with anxiety are so pronounced there could be a sex-specific cause. One possible explanation could be related to how women with anxiety are perceived. Women in their fifties die from suicide at a lower rate than men in their fifties and engage in self-harm at a lower rate than women in younger age groups.^2^^,^^22^^,^^23^ This could lead to underestimation of risk and need in this group. Alternatively, there could be an impact from the onset and completion of the menopause, which is known to be associated with symptoms of anxiety and depression.^24^ The higher rate seen for women in their fifties could be an impact of these more severe symptoms or rates could be driven by clinicians dismissing symptoms of anxiety and depression as a “natural” part of the menopause and therefore being transient and of less significance. Further work to understand the experiences of women in this age group and those of men aged 70 and over with anxiety conditions may help unpack the impact of these factors.

Most estimates of suicide rates are either from psychological autopsy studies with small sample sizes or cohort studies from psychiatric outpatient or inpatient settings, which encompasses people with only the most symptomatic presentations of mental health conditions.^3^^,^^4^^,^^25^

Comparing this study to those presented in a 2021 systematic review and meta-regression the diagnoses included under the heading of anxiety and depression differed from those presented here.^4^ Most studies estimating the rate of suicide in those with anxiety used a combination of all Diagnostic and Statistical Manual of Mental Disorders (DSM) III or DSM-IV anxiety diagnoses which includes Post-Traumatic Stress Disorder (PTSD) and Obsessive-Compulsive Disorder; neither of which were included in this study.^26^^,^^27^ The National Institute for Health and Care Excellence (NICE), which produces national guidelines on the management of physical and mental health conditions in England advises that the role of the primary care in PTSD is to diagnose and that “special referral [is] required for any further management”.^28^ It does not seem appropriate to combine PTSD with anxiety disorders in a primary care context when the management decisions around the diagnosis are so distinct from all other anxiety disorders. The meta-regression estimated that the relative risk of suicide for men with anxiety was 5·0 (95% CI 2·8–8·9) and for women with anxiety 4·8 (95% CI 2·7–8·6) compared to those without a diagnosis. Multiplying this relative risk by the suicide rate per 100,000 people per year in England in 2008 (the mid-year of this study) would be the equivalent estimated rate of approximately 73·2 (95% CI 39·5–135·4) for men and 21·2 (95% CI 11·3–40·6) for women—similar to the rates reported here (69·34 (95% CI 59·20–80·73) for men and 15·18 (95% CI 11·53–19·63) for women) despite methodological differences.^18^

In the same 2021 review dysthymia was analysed separately from major depressive disorder and was found to have the lowest relative risk of suicide of all mental disorders studied 4·11 (95% CI 2·09–8·09).^4^ Some diagnoses would have been applied retrospectively in the eleven psychological autopsy studies and people with symptoms of depression that were not severe enough to meet the criteria for major depressive disorder would have been excluded from that relative risk estimate. The distinction between dysthymia and major depressive disorder is not commonly made in UK primary care where dysthymia is not routinely used. The meta-regression estimated the relative risk of suicide in men with depression as 7·8 (95% CI 4·3–13·9) and for women with depression 7·5 (95% CI 4·2–13·5) compared to those without a diagnosis. Multiplying this relative risk by the suicide rate per 100,000 people per year in England in 2008 (the mid-year of this study) this would be the equivalent of approximately 114·4 (95% CI 61·6–211·7) for men and 33·0 (95% CI 17·6–63·5) for women—consistent with the rates reported here (115·85 (95% CI 107·60–124·58) for men and 22·83 (95% CI 19·95–26·00) for women).^18^

Another systematic review in 2021 found four studies that reported the rate of suicide in those with major depressive disorder and estimated the rate of suicide as 534·3 per 100,000 PYAR, with wide confidence intervals (95% CI 30·4–1448·7).^3^ Two of the studies were in a psychiatric inpatient setting and in another, 80% of individuals were recruited from a psychiatric inpatient setting. The rate reported in this meta-analysis far exceeds all rates reported in this study, which is expected given the difference in settings. These two meta-analyses compared the rates of suicide associated different conditions. They both reported that major depression was associated with a higher rate of suicide than observed for bipolar disorder or schizophrenia, although with wide confidence intervals. One review reported a relative risk (RR) of suicide for major depression as 7·6 (95% CI 4·3–13·6) compared to no diagnosis. This was higher than for bipolar disorder with a RR of 6·1 (95% CI 3·4–10·8) and for schizophrenia, with a RR of 6·0 (95% CI 3·3–10·7). In contrast, anxiety disorders were reported as having a RR of 4·9 (95% CI 2·7–8·7).^4^ The other review reported incidence rates per 100,000 person-years for major depression as 534·3 (95% CI 30·4–1448·7) compared with 237·0 (95% CI 159·9–328·5) for bipolar disorder and 352·2 (95% CI 239·3–485·7) for schizophrenia.^3^

We did not identify any comparable studies looking at suicide rates after diagnoses of mixed anxiety and depression.

The lack of correlation between deprivation and suicide in this study is consistent with the known association between deprivation and both common mental disorder diagnosis and suicide risk.^29^, ^30^, ^31^ By studying those with a diagnosis of a common mental disorder (in this case depression, anxiety, or mixed anxiety and depression), this has the effect of effectively stratifying by diagnosis and nullifying the association with suicide.

For clinicians and policymakers, understanding the rate of suicide in primary care attenders after a first diagnosis of depression, anxiety, or mixed anxiety and depression has implications that are distinct from understanding rates at either a general population level or in a secondary care setting. The far higher incidence of suicide for men relative to women should be of concern to clinicians, suggesting the need for additional attention for men with new diagnosis of depression and/or anxiety in primary care. Primary care has a central role in both the early management of common mental disorders and in suicide risk assessment. There is health economic evidence to suggest a benefit from the use of targeted active follow-up or cognitive behavioural therapy with improved identification of those most at risk of death after presenting to primary care.^32^ Clinicians in primary care diagnosing a person for the first time have less information available to them than those in secondary care, who will have information relating to the referral and an understanding that the presentation is severe enough to warrant review. This study provides valuable information to clinicians assessing suicide risk and to policymakers producing clinical and public health guidance.

Our findings highlight groups that may warrant additional attention after a first diagnosis of depression, anxiety, or mixed anxiety and depression. Notably, women experiencing anxiety for the first time in their fifties and men diagnosed with anxiety and depression for the first time aged 70 and over are at particularly high risk. These findings suggest a pressing need to better understand the experiences of these groups and how their risk is evaluated in a clinical setting.

This work also underscores the need for risk assessment strategies and crisis plans that are sensitive to the nuances of anxiety and depression as they present across the life-course. Personalised approaches to risk assessment and crisis planning could play a role in reducing the high rate of suicide seen in these groups.^33^

Due to the exploratory nature of these findings, and the width of confidence intervals, further study is required to repeat and refine the work. If corroborated, future research should aim to delineate the extent to which these deaths might be preventable through earlier diagnosis, more refined risk assessment, and modifications in clinical management. A deeper understanding of the lived experiences before and after a diagnosis will be critical in refining risk assessment strategies and informing the design of targeted interventions.

Furthermore, the potential misclassification of accidental deaths, especially among older women not traditionally deemed high-risk, warrants further investigation.

In conclusion, our study brings an exploration of factors affecting suicide rate in a primary care population newly diagnosed with depression, anxiety, or mixed anxiety and depression. With additional research we aim to improve our understanding of age-specific presentations, risk factors, and risk mitigations in this setting.

## Contributors

Conceptualisation: All authors.

Data access and verification: James Bailey, Irene Petersen.

Data curation: James Bailey.

Formal analysis: James Bailey.

Funding acquisition: All authors.

Investigation: All authors.

Methodology: All authors.

Project administration: All authors.

Writing – original draft: James Bailey.

Writing – review & editing: All authors.

## Data sharing statement

We are unable to share the data, which is available from CPRD.

## Declaration of interests

JB and this study were funded by an NIHR Doctoral Fellow [NIHR302551]. All other authors are supervisors of JB as part of this fellowship but do not receive direct funding as part of it.

PS has no interests to declare.

SF has received honoraria for speaking at a seminar on psychiatric aspects of epilepsy care (including suicide risk in epilepsy), has provided expert clinical reports for Coroner investigations into deaths in custody, is an expert panel member of the UK's Independent Panel on Deaths in Custody (an arms-length advisory non-departmental public body co-sponsored by the Ministry of Justice, the Home Office, and the Department of Health and Social Care).

IN has had grants funded by UKRI and NIHR on topics related to mental health but not directly related to the work on suicide as undertaken in this paper, has been paid by Barts Charity for providing expert advice on their funding panel, has been paid by India Alliance of BDT and Wellcome Trust for Chairing their Team Science Collaborations and the Clinical Research Centers grants committee, chairs a Data Safety Monitoring Committee for a RCT on management of the long COVID syndrome led by the London School of Hygiene and Tropical Medicine, Chairs the IA grants committee and is a member of the Barts Charity Funding Committee until December 2024.

IP is primary supervisor of JB's NIHR Doctoral Fellowship. IP has grants funded by NIHR on topics related to mental health but not directly related to the work on suicide as undertaken in this paper.

## Appendix A Supplementary data

Appendices 1–3
