# Supplementary material for: Incidence of suicide within two years of a first diagnosis of depression, anxiety, or mixed anxiety and depression: an exploratory cohort study in primary care using the Clinical Practice Research Datalink
Source: eClinicalMedicine. 2025 Aug 25;87:103441. doi: 10.1016/j.eclinm.2025.103441 (PMC12496219; doi:10.1016/j.eclinm.2025.103441)
Supplement: Appendices 1–3 [file mmc1.docx]

**Incidence of suicide within two years of a first diagnosis of depression, anxiety, or mixed anxiety and depression: an exploratory cohort study in primary care using the Clinical Practice Research Datalink**

**Table of Contents**

Appendix 1: Diagnostic codes for depression, anxiety, and mixed anxiety and depression………………………2

Appendix 2: ICD codes for suicide………………………………………………………………………………..7

Appendix 3: Rate and incidence rate ratio tables with accidental death codes included………………………….8

References……………………………………………………………………………………………..…………10

**Appendix 1: Diagnostic codes for depression, anxiety, and mixed anxiety and depression**

| **Term** | **Condition** |
| --- | --- |
| [X]Agoraphobia without history of panic disorder | anxiety |
| [X]Anxiety disorder, unspecified | anxiety |
| [X]Generalized anxiety disorder | anxiety |
| [X]Other anxiety disorders | anxiety |
| [X]Other mixed anxiety disorders | anxiety |
| [X]Other specified anxiety disorders | anxiety |
| [X]Panic disorder [episodic paroxysmal anxiety] | anxiety |
| [X]Panic disorder with agoraphobia | anxiety |
| Agoraphobia | anxiety |
| Agoraphobia | anxiety |
| Agoraphobia | anxiety |
| Agoraphobia with panic attacks | anxiety |
| Agoraphobia without history of panic disorder | anxiety |
| Anxiety disorder | anxiety |
| Chronic anxiety | anxiety |
| GAD - Generalized anxiety disorder | anxiety |
| Generalised anxiety disorder | anxiety |
| Generalized anxiety disorder | anxiety |
| Organic anxiety disorder | anxiety |
| Panic disorder | anxiety |
| Panic disorder with agoraphobia | anxiety |
| Panic disorder with agoraphobia AND mild panic attacks | anxiety |
| Panic disorder with agoraphobia AND moderate panic attacks | anxiety |
| Panic disorder with agoraphobia AND severe panic attacks | anxiety |
| Panic disorder without agoraphobia | anxiety |
| Panic disorder without agoraphobia with mild panic attacks | anxiety |
| Panic disorder without agoraphobia with moderate panic attacks | anxiety |
| Panic disorder without agoraphobia with panic attacks in partial remission | anxiety |
| Recurrent panic attacks | anxiety |
| Social anxiety disorder | anxiety |
| [RFC] Depression | depression |
| [RFC] Postnatal depression | depression |
| [X]Atypical depression | depression |
| [X]Depressive disorder NOS | depression |
| [X]Depressive episode, unspecified | depression |
| [X]Endogenous depression with psychotic symptoms | depression |
| [X]Endogenous depression without psychotic symptoms | depression |
| [X]Mild depression | depression |
| [X]Moderate depressive episode | depression |
| [X]Monopolar depression NOS | depression |
| [X]Other depressive episodes | depression |
| [X]Postnatal depression NOS | depression |
| [X]Postpartum depression NOS | depression |
| [X]Recurr depress disorder cur epi severe without psyc sympt | depression |
| [X]Recurr severe episodes/major depression+psychotic symptom | depression |
| [X]Recurr severe episodes/psychogenic depressive psychosis | depression |
| [X]Recurrent depress disorder cur epi severe with psyc symp | depression |
| [X]Recurrent depressive disorder | depression |
| [X]Recurrent depressive disorder, current episode mild | depression |
| [X]Recurrent depressive disorder, current episode moderate | depression |
| [X]Recurrent depressive disorder, unspecified | depression |
| [X]Recurrent episodes of depressive reaction | depression |
| [X]Recurrent episodes of psychogenic depression | depression |
| [X]Recurrent episodes of reactive depression | depression |
| [X]Recurrent major depressive episodes, severe, with psychosis, psychosis in remission | depression |
| [X]Recurrent severe episodes of psychotic depression | depression |
| [X]Seasonal depressive disorder | depression |
| [X]Severe depressive episode with psychotic symptoms | depression |
| [X]Severe depressive episode without psychotic symptoms | depression |
| [X]Single episode of depressive reaction | depression |
| [X]Single episode of major depression and psychotic symptoms | depression |
| [X]Single episode of masked depression NOS | depression |
| [X]Single episode of psychogenic depression | depression |
| [X]Single episode of psychogenic depressive psychosis | depression |
| [X]Single episode of psychotic depression | depression |
| [X]Single episode of reactive depression | depression |
| [X]Single episode of reactive depressive psychosis | depression |
| [X]Single episode vital depression w'out psychotic symptoms | depression |
| [X]Single major depressive episode, severe, with psychosis, psychosis in remission | depression |
| Acute depression | depression |
| Agitated depression | depression |
| Agitated depression | depression |
| Antenatal depression | depression |
| Antenatal depression | depression |
| Atypical depressive disorder | depression |
| Chronic depression | depression |
| Chronic major depressive disorder, single episode | depression |
| Chronic recurrent major depressive disorder | depression |
| Depressed | depression |
| Depressed | depression |
| Depression | depression |
| Depression | depression |
| Depression | depression |
| Depression annual review | depression |
| Depression confirmed | depression |
| Depression management programme | depression |
| Depression medication review | depression |
| Depression NOS | depression |
| Depressive disorder | depression |
| Depressive episode | depression |
| Depressive episode | depression |
| Depressive illness | depression |
| Depressive psychoses | depression |
| Dysthymia | depression |
| Endogenous depression | depression |
| Endogenous depression - recurrent | depression |
| Endogenous depression first episode | depression |
| Endogenous depression first episode | depression |
| Episode of depression | depression |
| Follow up for depression | depression |
| Major depression | depression |
| Major depression with psychotic features | depression |
| Major depression, single episode | depression |
| Major depressive disorder | depression |
| Major depressive disorder, single episode | depression |
| Major depressive disorder, single episode with catatonic features | depression |
| Major depressive disorder, single episode with postpartum onset | depression |
| Major depressive disorder, single episode, severe with psychotic features | depression |
| Masked depression | depression |
| Maternal postnatal depression | depression |
| Mild depression | depression |
| Mild depression | depression |
| Mild depressive episode, with somatic syndrome | depression |
| Mild depressive episode, without somatic syndrome | depression |
| Mild major depression | depression |
| Mild major depression, single episode | depression |
| Mild postnatal depression | depression |
| Mild recurrent major depression | depression |
| Minimal major depression single episode | depression |
| Minor depressive disorder | depression |
| Moderate depression | depression |
| Moderate depression | depression |
| Moderate depressive episode, with somatic syndrome | depression |
| Moderate depressive episode, without somatic syndrome | depression |
| Moderate major depression | depression |
| Moderate major depression, single episode | depression |
| Moderate recurrent major depression | depression |
| Moderately severe depression | depression |
| Moderately severe major depression single episode | depression |
| On depression register | depression |
| Organic depressive disorder | depression |
| Other recurrent mood affective disorders, recurrent brief depressive disorder | depression |
| Perinatal depression | depression |
| Post natal depression | depression |
| Postpartum depression | depression |
| Psychotic depression | depression |
| Psychotic reactive depression | depression |
| Puerperal depression | depression |
| Reactive (neurotic) depression | depression |
| Reactive depression | depression |
| Reactive depression (situational) | depression |
| Reactive depression, prolonged single episode | depression |
| Reactive depressive psychosis | depression |
| Reactive depressive psychosis, single episode | depression |
| Recurrent brief depressive disorder | depression |
| Recurrent depression | depression |
| Recurrent depression with current moderate episode | depression |
| Recurrent depression with current severe episode and psychotic features | depression |
| Recurrent depressive disorder, current episode mild, with somatic syndrome | depression |
| Recurrent depressive disorder, current episode moderate, with somatic syndrome | depression |
| Recurrent depressive disorder, current episode moderate, without somatic syndrome | depression |
| Recurrent major depression | depression |
| Recurrent major depressive disorder | depression |
| Recurrent major depressive episode NOS | depression |
| Recurrent major depressive episodes | depression |
| Recurrent major depressive episodes, mild | depression |
| Recurrent major depressive episodes, moderate | depression |
| Recurrent major depressive episodes, severe | depression |
| Recurrent major depressive episodes, severe, with psychosis | depression |
| Recurrent major depressive episodes, severe, with psychosis, psychosis in remission | depression |
| Recurrent major depressive episodes, unspecified | depression |
| Recurrent reactive depressive episodes, severe, with psychosis | depression |
| Referral for guided self-help for depression | depression |
| SAD - Seasonal affective disorder | depression |
| Seasonal affective disorder | depression |
| Severe depression | depression |
| Severe depression | depression |
| Severe major depression with psychotic features | depression |
| Severe major depression without psychotic features | depression |
| Severe major depression, single episode | depression |
| Severe major depression, single episode, with psychotic features | depression |
| Severe major depression, single episode, with psychotic features, mood-congruent | depression |
| Severe major depression, single episode, without psychotic features | depression |
| Severe postnatal depression | depression |
| Severe recurrent major depression with psychotic features | depression |
| Severe recurrent major depression without psychotic features | depression |
| Single major depressive episode NOS | depression |
| Single major depressive episode, severe, with psychosis | depression |
| Single major depressive episode, severe, with psychosis, psychosis in remission | depression |
| Single major depressive episode, unspecified | depression |
| [X]Mild anxiety depression | mixed |
| [X]Mixed anxiety and depressive disorder | mixed |
| [X]Persistant anxiety depression | mixed |
| Anxiety depression | mixed |
| Mild major depressive disorder co-occurrent with anxiety single episode | mixed |
| Mixed anxiety and depressive disorder | mixed |
| Mixed anxiety and depressive reaction | mixed |
| Moderate major depressive disorder co-occurrent with anxiety single episode | mixed |
| Recurrent moderate major depressive disorder co-occurrent with anxiety | mixed |
| Recurrent severe major depressive disorder co-occurrent with anxiety | mixed |
| Severe major depressive disorder co-occurrent with anxiety single episode | mixed |

**Appendix 2: ICD codes for suicide**

Death from suicide was defined to be consistent with the ONS mortality statistics methodology.^1^ These included all International Classification of Diseases (ICD) 9th Revision and 10th Revision codes for “intentional self-harm” (ICD-10: X60 to X84, ICD-9: E950 to E959) and “injury/poisoning of undetermined intent” (ICD-10: Y10 to Y34, ICD-9 E980 to E989) excluding Y33·9 and E988·8. If one of these codes was present at any level of underlying cause of death on the ONS Death Registry, then the death was be considered a suicide.

**Appendix 3: Rate and incidence rate ratio tables with accidental death codes included**

|  | **Depression** | | | | **Anxiety** | | | | **Mixed anxiety and depression** | | | |
| --- | --- | --- | --- | --- | --- | --- | --- | --- | --- | --- | --- | --- |
|  | **Men** | | **Women** | | **Men** | | **Women** | | **Men** | | **Women** | |
|  | **Rate** | **(95% CI)** | **Rate** | **(95% CI)** | **Rate** | **(95% CI)** | **Rate** | **(95% CI)** | **Rate** | **(95% CI)** | **Rate** | **(95% CI)** |
| **Total** | 140·80 | (131·68 - 150·39) | 30·60 | (27·25 - 34·25) | 86·89 | (75·48 - 99·53) | 20·94 | (16·60 - 26·06) | 131·48 | (114·82 - 149·87) | 34·28 | (27·53 - 42·18) |
|  |  |  |  |  |  |  |  |  |  |  |  |  |
| **18-29** | 114·39 | (97·65 - 133·17) | 23·45 | (18·02 - 30·00) | 61·16 | (44·44 – 82·10) | 14·04 | (8·03 - 22·81) | 87·75 | (62·69 - 119·49) | 29·03 | (17·97 - 44·37) |
| **30-39** | 147·27 | (127·18 - 169·64) | 23·43 | (17·55 – 30·65) | 65·75 | (45·53 - 91·88) | 10·63 | (4·59 - 20·95) | 149·83 | (114·06 - 193·27) | 19·30 | (9·63 - 34·53) |
| **40-49** | 148·57 | (128·35 - 171·08) | 37·32 | (28·98 - 47·31) | 127·70 | (96·46 - 165·83) | 20·88 | (11·12 - 35·70) | 121·14 | (88·02 - 162·62) | 47·83 | (30·65 - 71·17) |
| **50-59** | 146·39 | (123·75 - 171·96) | 32·03 | (23·08 - 43·29) | 125·31 | (89·52 – 170·63) | 39·89 | (24·01 - 62·29) | 155·93 | (111·90 - 211·54) | 37·68 | (20·60 - 63·22) |
| **60-69** | 130·80 | (103·05 - 163·71) | 33·53 | (21·48 - 49·89) | 64·25 | (34·21 - 109·86) | 26·09 | (11·93 - 49·52) | 151·23 | (91·05 - 236·17) | 51·49 | (24·69 - 94·69) |
| **70+** | 171·73 | (141·27 - 206·81) | 48·06 | (36·00 - 62·87) | 106·47 | (65·90 - 162·74) | 30·96 | (17·33 - 51·07) | 205·31 | (127·09 - 313·84) | 38·19 | (17·46 - 72·49) |
|  |  |  |  |  |  |  |  |  |  |  |  |  |
| **1 (least deprived)** | 148·82 | (126·85 - 173·50) | 37·41 | (29·05 - 47·42) | 85·08 | (60·78 - 115·86) | 22·50 | (13·33 - 35·56) | 146·84 | (105·83 - 198·48) | 20·13 | (9·20 - 38·20) |
| **2** | 127·96 | (108·37 - 150·08) | 29·38 | (22·19 - 38·15) | 90·18 | (65·26 - 121·47) | 13·98 | (6·98 - 25·01) | 128·36 | (91·70 - 174·79) | 34·53 | (20·11 - 55·28) |
| **3** | 141·82 | (121·12 - 165·03) | 23·46 | (17·04 - 31·49) | 81·62 | (57·47 - 112·51) | 27·59 | (16·85 - 42·61) | 117·40 | (82·66 - 161·82) | 44·85 | (28·11 - 67·91) |
| **4** | 143·54 | (123·95 - 165·34) | 30·47 | (23·41 - 38·98) | 91·82 | (66·72 - 123·27) | 22·69 | (13·22 - 36·33) | 126·48 | (92·26 - 169·24) | 30·94 | (18·03 - 49·54) |
| **5 (most deprived)** | 141·82 | (123·41 - 162·20) | 32·24 | (25·23 - 40·60) | 85·53 | (62·15 - 114·82) | 18·45 | (10·08 - 30·95) | 137·91 | (105·24 - 177·51) | 38·88 | (24·91 - 57·85) |

|  |  |  |  |  |  |  |  |  |  |  |  |  |
| --- | --- | --- | --- | --- | --- | --- | --- | --- | --- | --- | --- | --- |
|  | **Depression** | | | | **Anxiety** | | | | **Mixed anxiety and depression** | | | |
|  | **Men** | | **Women** | | **Men** | | **Women** | | **Men** | | **Women** | |
|  | **aIRR** | **(95% CI)** | **aIRR** | **(95% CI)** | **aIRR** | **(95% CI)** | **aIRR** | **(95% CI)** | **aIRR** | **(95% CI)** | **aIRR** | **(95% CI)** |
| **Total** | 4·53 | (3·97 - 5·16) | ref. | | 4·16 | (3·21 - 5·39) | ref. | | 3·83 | (2·99 - 4·90) | ref. | |
|  |  |  |  |  |  |  |  |  |  |  |  |  |
| **18-29** | ref. | | ref. | | ref. | | ref. | | ref. | | ref. | |
| **30-39** | 1·28 | (1·04 - 1·58) | 1·00 | (0·69 - 1·44) | 1·07 | (0·69 - 1·68) | 0·76 | (0·32 - 1·77) | 1·71 | (1·14 - 2·55) | 0·67 | (0·32 - 1·40) |
| **40-49** | 1·30 | (1·06 - 1·61) | 1·58 | (1·12 - 2·23) | 2·10 | (1·41 - 3·11) | 1·50 | (0·72 - 3·12) | 1·39 | (0·90 - 2·13) | 1·69 | (0·94 - 3·05) |
| **50-59** | 1·29 | (1·03 - 1·61) | 1·36 | (0·92 - 2·02) | 2·06 | (1·34 - 3·17) | 2·87 | (1·47 - 5·58) | 1·79 | (1·16 - 2·77) | 1·34 | (0·68 - 2·64) |
| **60-69** | 1·16 | (0·88 - 1·52) | 1·43 | (0·90 - 2·30) | 1·06 | (0·57 - 1·96) | 1·88 | (0·83 - 4·25) | 1·74 | (1·01 - 3·02) | 1·83 | (0·86 - 3·89) |
| **70+** | 1·52 | (1·20 - 1·94) | 2·06 | (1·43 - 2·97) | 1·76 | (1·05 - 2·97) | 2·23 | (1·10 - 4·53) | 2·38 | (1·40 - 4·05) | 1·37 | (0·63 - 3·00) |
|  |  |  |  |  |  |  |  |  |  |  |  |  |
| **1 (least deprived)** | 1·03 | (0·84 - 1·26) | 1·10 | (0·79 - 1·54) | 0·94 | (0·61 - 1·44) | 1·11 | (0·55 - 2·24) | 1·00 | (0·68 - 1·49) | 0·49 | (0·23 - 1·05) |
| **2** | 0·89 | (0·72 - 1·09) | 0·87 | (0·62 - 1·24) | 1·01 | (0·66 - 1·54) | 0·71 | (0·32 - 1·56) | 0·88 | (0·59 - 1·32) | 0·85 | (0·46 - 1·58) |
| **3** | 0·99 | (0·81 - 1·21) | 0·71 | (0·49 - 1·03) | 0·93 | (0·60 - 1·44) | 1·43 | (0·72 - 2·84) | 0·82 | (0·54 - 1·23) | 1·12 | (0·63 - 2·00) |
| **4** | 1·00 | (0·83 - 1·22) | 0·93 | (0·66 - 1·31) | 1·07 | (0·70 - 1·62) | 1·21 | (0·60 - 2·46) | 0·90 | (0·61 - 1·32) | 0·78 | (0·42 - 1·46) |
| **5 (most deprived)** | ref. | | ref. | | ref. | | ref. | | ref. | | ref. | |

**Reference**

1. Office for National Statistics. Suicide rates in the UK: quality and methodology information. London: ONS, 2019.
